# Effectiveness of international border control measures during the COVID-19 pandemic: a narrative synthesis of published systematic reviews

**DOI:** 10.1098/rsta.2023.0134

**Published:** 2023-10-09

**Authors:** Karen Ann Grépin, John Aston, Jacob Burns

**Affiliations:** ^1^ School of Public Health, University of Hong Kong Faculty of Medicine, Pokfulam, Hong Kong; ^2^ Statistical Laboratory, University of Cambridge, Cambridge, CB3 0WB, UK; ^3^ Ludwig-Maximilians University, Munich, 81377, Germany

**Keywords:** non-pharmaceutical interventions, travel, COVID-19, epidemiology, systematic review

## Abstract

The effectiveness of international border control measures during the COVID-19 pandemic is not well understood. Using a narrative synthesis approach to published systematic reviews, we synthesized the evidence from both modelling and observational studies on the effects of border control measures on domestic transmission of the virus. We find that symptomatic screening measures were not particularly effective, but that diagnostic-based screening methods were more effective at identifying infected travellers. Targeted travel restrictions levied against travellers from Wuhan were likely temporarily effective but insufficient to stop the exportation of the virus to the rest of the world. Quarantine of inbound travellers was also likely effective at reducing transmission, but only with relatively long quarantine periods, and came with important economic and social effects. There is little evidence that most travel restrictions, including border closure and those implemented to stop the introduction of new variants of concern, were particularly effective. Border control measures played an important role in former elimination locations but only when coupled with strong domestic public health measures. In future outbreaks, if border control measures are to be adopted, they should be seen as part of a broader strategy that includes other non-pharmaceutical interventions.

This article is part of the theme issue 'The effectiveness of non-pharmaceutical interventions on the COVID-19 pandemic: the evidence'.

## 1. Introduction

The use of international border control measures to reduce the transmission of infectious diseases has a long history, dating back to the quarantine of maritime travellers during medieval times and more recently in response to Severe Acute Respiratory Syndrome, Middle East Respiratory Syndrome, tuberculosis, and Ebola virus disease. However, the scale and breadth of border control measures adopted during the COVID-19 pandemic are unprecedented; by early 2020, all countries had adopted some form of international border control measure, and some countries maintained the use of such measures into 2023. In addition, a more diverse set of measures was adopted during the pandemic than before, such as complete border closures, vaccination requirements for incoming travellers and centralized quarantine for inbound travellers in hotels.

Prior to the emergence of COVID-19, it was assumed that the effectiveness of border control measures was low and, thus, that they are generally not worth implementing. The role of international border control measures in reducing transmission during the COVID-19 pandemic remains unclear and evaluating the effectiveness of such measures is challenging. Border control measures have been broadly defined as ‘action taken to control movement of people (travel) or trade across two or more jurisdictions with the stated intent of achieving a health goal’ [1]. Specifically, border control measures include travel advice; screening of travellers for infection, including both symptomatic and diagnostic-based screening methods; travel restrictions on specific types of travellers; complete border closure; and quarantine of incoming travellers. While these are all referred to as border control measures, they do different things, have different aims and operate through different mechanisms; thus, they are likely to have very different levels of effectiveness and should be evaluated independently. Most countries implemented a mix of border control measures during the pandemic, usually alongside other non-pharmaceutical interventions (NPIs), further complicating the evaluation of their effectiveness in practice. In addition, the effectiveness of border control measures is likely to vary depending on the prevalence and incidence of the virus in both the sending and receiving jurisdictions as well as the mix of variants in circulation. Therefore, it is also important to consider the implementation timing and context of measures in their evaluation.

Without any intervention, we can reasonably assume that the prevalence among international travellers reflects the prevalence in the country from which a traveller originates [2]. More precisely, the number of infected travellers entering a new country is a function of the prevalence among travellers and the number of travellers from the originating country. An infected traveller may transmit the virus to susceptible people locally both while travelling and upon arrival at the destination country, thus contributing to the domestic outbreak. However, once an imported case leads to local transmission, future transmission will be heavily influenced by local levels of immunity as well as the mix of local NPIs in place.

Theoretically, border control measures could reduce transmission into a country through at least four channels:

1. Reducing the number of infected people that cross the border in the first place, which can happen either by reducing the total number of travellers or making it less likely that infected people travel.
2. Reducing the risk of infection during international travel, which includes measures to reduce the likelihood that infected patients board a plane and transmit the infection to others. Measures such as mandatory masking or circulation of air on planes would also belong to this category; however, they are excluded from this synthesis as they overlap with environmental controls.
3. Reducing the likelihood of an imported case transmitting the virus to the susceptible local population, typically by isolating or quarantining travellers from local populations until the risk of transmission is low or zero.
4. Otherwise reducing the risk of an infected case affecting disease dynamics in the local population. For example, travel measures could theoretically stop the importation of specific variants of the virus.

As of early 2023, several published systematic reviews, including three written by the authors of this paper, have examined the evidence on the effectiveness of border control measures. The first set of rapid reviews investigated the effectiveness of border control measures during the early stages of the pandemic (i.e. through June 2020) and focused largely on the effects of targeted travel restrictions imposed on travellers from Wuhan and other parts of China, as well as those from Iran, Italy and South Korea, on containment, as well as the effects of the other types of border control measures that were widely adopted in early 2020, such as symptomatic screening. Subsequent systematic reviews lengthened the time periods investigated and identified evidence on additional measures, including border closures, diagnostic-based screening and quarantine. In the absence of a synthesis of these reviews, it remains challenging to appreciate fully what has been learned regarding the effectiveness of border control measures.

To address this gap, we aim to synthesize the best available systematic review evidence on the effectiveness of international border control measures during the COVID-19 pandemic using a narrative synthesis approach, which is a method that enables the integration of evidence from multiple sources using a textual approach to summarize the available evidence. Specifically, our synthesis aims to answer the following research questions:

1. What forms of border control measures, if any, have been shown to be effective in reducing the transmission of COVID-19 during the pandemic?
2. In which locations, if any, were these measures effective?
3. At what time were such measures effective?

## 2. Methodology

To answer these questions, a three-step process was followed. First, the evidence collected from existing systematic reviews of international border control measures was brought together using a narrative synthesis approach. Second, evidence from additional studies that were too recent to have been included in the published reviews is discussed. We also discuss other types of studies that provide additional insights into the effectiveness of border control measures, because the studies included in the systematic reviews may not have captured all evidence of the effectiveness of border controls. Third, we aggregate the evidence from observational studies to further understand the real-world effectiveness of border control measures during the pandemic.

In aiming to identify all relevant evidence, we chose not to limit the choice of populations, interventions, comparisons and outcomes (PICO). Specifically, all systematic reviews that assessed transmission-related outcomes of any of the international border control measures described above were considered, including evidence from both modelling and observational studies conducted during the COVID-19 pandemic.

A key challenge in measuring the effectiveness of international border control measures is the lack of a standardized definition and typology to fully describe the universe of measures adopted during the pandemic [1]. A detailed review of the types of measures adopted during the pandemic is beyond the scope of the present study, which focuses on border control measures that meet the following criteria:

— measures aimed at controlling the transmission of COVID-19 across a border;
— commonly used border control measures (i.e. those adopted by many countries during the COVID-19 pandemic);
— measures that are non-overlapping with other NPIs included in the Royal Society evidence review (e.g. masking of travellers or environmental controls enforced on modes of transportation).

This allowed us to focus on the following border control measures:

— Border closure, which is defined as the complete restriction of both inbound and outbound travellers via a specific port (i.e. air, land or sea) or all ports.
— Travel restrictions, which are partial forms of border closure aimed only at specific jurisdictions (e.g. a flight route between two locations) or specific types of travellers (e.g. those originating/transiting in specific countries or non-citizens). There is a fine line between a partial and complete border restriction and thus at times the identified systematic reviews grouped border closures and travel restrictions together.
— Screening of travellers before travel or upon arrival, which includes symptomatic screening (e.g. based on fever or other symptoms), exposure-based screening (e.g. based on health questionnaires) and diagnostic testing (e.g. a PCR or rapid antigen test or antibody tests). Countries also allowed exemptions to rules for vaccinated or recently infected travellers, which are akin to screening based on immunological status, so we included them under the screening category. Usually, screening is also paired with the isolation or quarantine of people who test positive or are deemed high risk for the virus, but this varies by international context.
— Quarantine, which is the physical separation of travellers not known to be infected from the rest of the public. Isolation refers to the physical separation of travellers known to be infected. However, the terms quarantine and isolation are often used interchangeably in both research and practice. Quarantine can be carried out at various locations (e.g. at home, in a hotel or in a specialized facility) and can vary in duration. It can also be performed in combination with other measures, such as diagnostic testing, to assess infection status.

Other forms of travel measures exist (e.g. travel advice), but there is insufficient evidence in the literature on their use during the COVID-19 pandemic and so they are not a focus of this synthesis. While not all border control measures were directed towards international borders—for example, several jurisdictions in China, Canada and Australia also imposed subnational border control measures—this review mainly focuses on the effectiveness of international border control measures. However, subnational evidence is included if it otherwise met the inclusion criteria of the systematic reviews.

In the case of border control measures, five published systematic reviews have summarized evidence on their effectiveness in affecting transmission during the COVID-19 pandemic [3–7]. There is also another systematic review in progress by Burns *et al*. [8]. These systematic reviews were identified through an extensive search of Google Scholar using the terms ‘review’, either ‘covid’ or ‘SARS-Cov-2’, and either of the words ‘travel’ or ‘border’. We also relied upon the expertise of the authors of this synthesis, who have collectively authored three of the five published systematic reviews and thus were familiar with the literature. Systematic reviews related to narrower topics, such as diagnostic testing of travellers, were excluded, as the travel components of these reviews were already captured in the reviews identified above. Systematic reviews that evaluated border control measures as part of a broader evaluation of multiple types of NPIs were also excluded, as they had a high degree of overlap with the existing systematic reviews. We also excluded reviews that were related to transmission of the virus during specific modes of travel (e.g. on aeroplanes or cruise ships), as these were generally not related to the use of specific border control measures but were more focused on environmental control measures. In addition, forward citation searching was used to identify any newer articles which cited Burns *et al*. [5], the most highly cited of the existing systematic reviews, but were not included in their update currently underway and would meet inclusion criteria consistent with the existing systematic reviews.

The reviews are synthesized in the order in which they were published, allowing us to highlight the new findings in each subsequent review, note any discrepancies with previous findings, and determine if any consensus had emerged in the literature. Synthesis without meta-analysis (SWiM) guidelines were used to report the narrative synthesis [9]. The SwiM guidelines were developed to report on reviews of quantitative studies for which meta-analysis is either not possible, not appropriate, or both. This approach is appropriate for the present synthesis given the heterogeneous nature of the interventions, the wide range of outcome measures, and the diverse geographical contexts of the studies identified within and across the existing systematic reviews. Specifically, even within each type of individual border control measure evaluated, the context varied greatly in terms of the timing and enforcement of the measures, as well as the underlying COVID-19 prevalence and additional NPIs in place. Additionally, very different evaluation methods were used in the underlying studies.

The main features of the identified systematic reviews included in this synthesis are summarized in table 1. For each review, the types of measures evaluated, main study designs adopted, sources used, inclusion and exclusion criteria applied, and quality assessment tools used are summarized. The study period of published articles for each review is also noted. While the overall approach taken by most of the reviews was similar, they differed in terms of the time periods they examined, whether they investigated subnational measures or not, and the tools used to assess the quality of evidence. The reviews summarized articles published from the start of the pandemic through June 2022. The forthcoming Burns *et al*. [8] systematic review captured studies through late 2022, while the forward citations were considered through the drafting of this article in early 2023. With regard to certainty of evidence, since we are building on existing systematic reviews, we report using the pre-existing quality assessments. The specific methods used to assess varied by review are summarized in table 1.

**Table 1.** Design features of existing systematic reviews.

review	measures evaluated	study design	sources	inclusion criteria	exclusion criteria	quality assessment tools	dates of relevant articles
Grépin *et al*. [6]	symptomatic screening, travel restrictions, border closure and quarantine	rapid review	PubMed, BioRxiv/MedRxiv, WHO COVID-19 Research Database	COVID-19; domestic or international measures; modelled or observational; any outcome	news reports, review articles, commentaries or editorials	proprietary scoring system: low, medium and high	start of the pandemic to 1 June 2020
Burns *et al*. [4]	border closures, travel restrictions, screening and quarantine	Cochrane guidance for rapid reviews	MEDLINE, Embase and COVID-19-specific databases, including the WHO COVID-19 Research Database, the Cochrane COVID-19 Study Register and the CDC COVID-19 Research Database	COVID-19/SARS/MERS; international measures only; experimental, quasi-experimental, observational and modelling studies; must have quantitative impact	case reports, non-quantitative studies, diagnostic studies, non-empirical studies, systematic reviews and conference abstracts; subnational studies	ROBINS-I, QUADAS-2 and a proprietary tool for modelling studies	start of the pandemic to 26 June 2020
Bou-Karroum *et al*. [3]	screening, quarantine, travel restrictions and border closure	Cochrane	MEDLINE, PubMed, Embase, Cochrane Central Register, WHO COVID-19 Global Literature Database, Epistemonikos and the Norwegian Institute of Public Health's live map of COVID-19 evidence	COVID-19; international and subnational; randomized controlled trials, non-randomized studies, modelling studies, qualitative studies, research letters and abstracts	preprints, editorials, letters to the editors, commentaries and correspondence	GRADE and EVIDEM framework	start of the pandemic to end of December 2020
Burns *et al*. [5]	border closures, travel restrictions, screening (symptomatic and diagnostic), quarantine and combination of measures	Cochrane guidance for rapid reviews	MEDLINE, Embase, Cochrane COVID-19 study register, WHO COVID-19 Global Literature Database	COVID-19; international only; experimental and quasi-experimental studies, observational studies, modelling studies; must provide quantitative evidence	case reports, non-quantitative studies, diagnostic studies, non-empirical studies, systematic reviews and conference abstracts; subnational studies	ROBINS-I, QUADAS-2 and proprietary tool for modelling studies	start of the pandemic to 13 November 2020
Hohlfeld *et al*. [7]	air travel measures only, including quarantine, diagnostic screening or a combination of both types of measures	Cochrane	Scopus, PubMed, Cochrane COVID-19 database, WHO COVID-19 Global Literature Database, MedRXiv/BioRXiv	COVID-19; modelled and observational studies; epidemiological outcomes	case reports, case series, opinion papers, editorials, study protocols and trial registries; modelled studies were excluded in favour of observational and ecological studies	ROBINS-I and GRADE	1 November 2020 to 6 June 2022

Table 2 summarizes the additional features of the identified systematic reviews. Some but not all of the reviews were designed to be non-overlapping with previous reviews, so there are duplications of studies across reviews. We also pooled all of the studies reviewed in any of the published systematic reviews. In total, 135 studies were identified across the reviews. Eighty-eight of these studies were included in only one of the reviews (65%), while the remainder had been included in two or more of the reviews. Only 43 (32%) of the studies were observational, while the rest were modelled.

**Table 2.** Additional features of existing systematic reviews.

review	outcomes evaluated^a^	COVID-19 studies included (unique to review)	observational/ modelled	international/ subnational/ hypothetical	geographical coverage of studies	travel restrictions/ border closures^b^	screening	quarantine
Grépin *et al*. [6]	cases imported/exported, probability of an outbreak, delay to major outbreak, time to peak, risk of imported/exported cases, arrival time of first case, transmission, total cases/deaths, cases detected	29 (14)	3/26	12/16/1	China, Australia, European Union, Hong Kong SAR, India, Israel, Japan, South Korea, Thailand	26	3	2
Burns *et al*. [4]	cases avoided, cases detected, shift in epidemic development; secondary outcomes	25 (0)	8/17	22/0/3	China, Australia, Austria, Belgium, Canada, Denmark, France, Finland, Germany, Greece, India, Italy, Japan, New Zealand, Norway, Singapore, South Korea, Spain, Sweden, Switzerland, Thailand, UK, USA	11	9	1
Bou-Karroum *et al*. [3]	cases avoided, cases detected, positivity rate, change in outbreak dynamics, transmission, mortality rates; secondary outcomes	64 (32)	15/49	43/13/8	China, Argentina, Australia, Austria, Belgium, Brunei, Canada, Cyprus, Denmark, EU, France, Germany, Hong Kong SAR, India, Italy, Kazakhstan, Japan, Lebanon, Netherlands, Saudi Arabia, Singapore, South Korea, Spain, Sweden, Switzerland, Taiwan, Thailand, USA, UK, Vietnam	48	5	9
Burns *et al*. [5]	cases avoided, cases detected, shift in epidemic development; secondary outcomes	62 (25)	14/48	56/1/5	China, Australia, Austria, Bahrain, Belgium, Brazil, Brunei, Canada, Denmark, Ecuador, EU, France, Finland, Germany, Greece, Hong Kong SAR, India, Iran, Italy, Ireland, Japan, Kenya, Lebanon, Macao SAR, Malaysia, Mauritius, Netherlands, New Zealand, Norway, Portugal, Russia, Saudi Arabia, Singapore, South Africa, South Korea, Spain, Sweden, Switzerland, Taiwan, Thailand, Turkey, UK, USA, Vietnam	30	28	28
Hohlfeld *et al*. [7]	cases avoided, change in outbreak dynamics, cases detected; secondary outcomes	22 (17)	13/9	22/0/0	China, Afghanistan, Australia, Bahrain, Canada, French Polynesia, Italy, Japan, Madagascar, New Zealand, Singapore, South Korea, Spain, UK, USA	1	19	16

^a^Although these reviews included secondary outcomes, those outcomes were not considered in this knowledge synthesis.

^b^Studies generally operationalized border closures and travel restrictions in a very similar manner, i.e. through some reduction in expected incoming travellers. Given this, we feel that these studies do not allow the assessment of these two types of measure separately from one another, so we have collapsed the two categories into one and included all studies under the combined category.

Studies were also grouped according to intervention type. Since many modelling studies included border closure as the most restrictive form of travel restriction, border closure and travel restrictions were grouped together. Similarly, many studies, especially observational ones, investigated both quarantine and screening as these two measures were frequently implemented together. Subsequently, studies were grouped by whether they were modelling or observational studies. While we prioritize evidence from observational studies in our analysis, modelling studies are an important part of the evidence base, since for some interventions, especially quarantine, most of the understanding was derived from modelling studies.

There was a great deal of variety in specific epidemiological outcomes investigated (table 2), both within interventions and across interventions (e.g. cases avoided for travel restrictions and cases detected for quarantine measures). Initially, only a few geographical areas were the focus of investigation, but more geographical contexts were investigated in later reviews.

In table 3, we summarize the features of the observational studies included in the reviews. Among the 43 observational studies, most of the evidence comes from studies of screening or quarantine, especially both interventions in combination. Only 12 studies investigated any form of travel restriction, and none investigated border closure alone. Most of the studies evaluated national-level interventions, although there were a few subnational studies of the effects of the Wuhan travel restrictions within mainland China. Almost all the observational studies come from the early phases of the pandemic, with 65% and 85% of them focusing on study periods that were complete by the end of April and June 2020, respectively. There was less geographical coverage among the observational studies, and many of the former ‘zero-COVID’ or elimination locations (e.g. Australia, China, Hong Kong, Japan, Singapore) are over-represented in the studies.

**Table 3.** Summary of features of observational studies.

interventions evaluated	*n*	percentage of total
*alone*		
travel restrictions	10	23%
quarantine	2	5%
screening	11	26%
*in combination*		
travel restrictions and quarantine	1	2%
quarantine and screening	16	37%
*in combination but separately*		
travel restrictions and screening	1	2%
quarantine and screening	2	5%
studies with any evidence		
travel restrictions	12	28%
quarantine	21	49%
screening	29	67%
level of intervention		
international	40	93%
subnational	3^a^	7%
study period		
before end of April 2020	28	65%
before end of June 2020^b^	35	81%
geographical coverage	locations	
travel restrictions	China, Australia, Hong Kong SAR, Latin American countries, USA	
quarantine	China, Afghanistan, Australia, Bahrain, Brunei, Canada, France, Germany, Greece, Hong Kong SAR, Japan, Macao SAR, Malaysia, Singapore, South Korea, Vietnam	
		
screening	China, Australia, Bahrain, Brunei, Canada, France, French Polynesia, Germany, Greece, Italy, Japan, Macao SAR, Madagascar, Malaysia, Saudi Arabia, Singapore, South Korea, Spain, Thailand, UK, USA	
		

^a^All of these studies were investigations of the Wuhan travel measures.

^b^One study ran through 5 July 2020 and was counted here.

## 3. Narrative synthesis

### (a) Grépin *et al*. [6]

This early rapid review examined the effectiveness of border control measures in controlling the spread of COVID-19 during the early phase of the pandemic (studies or preprints available as of 1 June 2020). It identified 29 unique articles that investigated the effects of symptomatic screening, travel restrictions and broader forms of border control measures on epidemiological outcomes, including international (*n* = 13) and subnational border control measures adopted within China (*n* = 17). One study investigated both international and subnational border control measures. All but one of these early studies were modelling studies.

The key finding of this review was that outbound travel restrictions imposed on travellers from Wuhan (23 January 2020) and Hubei province (24 January 2020) were effective in reducing the number of cases exported to other places in China and internationally. It is estimated that 70–80% of exported cases were prevented in the weeks following the implementation of these measures. Most studies also found that earlier implementation of measures could improve effectiveness. One study estimated that by the time the Wuhan measures had been adopted, the virus had already been seeded throughout the country, and thus the Wuhan measures only had a small effect on delaying epidemics (i.e. by a few days) within China. However, the effects of the same measures were larger at the international level but became less effective over time as other Chinese cities became the major source of exported cases.

Evidence on the effectiveness of additional border control measures adopted by other jurisdictions on top of the Wuhan measures is less clear. Most of the studies in this review investigated targeted border control measures against Chinese travellers (flight suspensions and/or travel bans). One modelling study found that the additional measures adopted by other countries against Chinese travellers reduced the number of exported cases only by approximately a few hundred through the end of February 2020 (relative to approx. 80 000 confirmed cases in China by the end of February 2020), partially because the Wuhan measures themselves were highly effective. Two additional studies investigated the travel ban implemented by Australia on Chinese travellers, which started on 2 February 2020. Both studies concluded that these restrictions, which were levied only against Chinese travellers from China, reduced the number of imported cases, although they differed in whether they believed it was a few hundred or a few thousand averted cases.

This review also identified three modelling studies that investigated symptomatic screening and concluded that the use of such measures was unlikely to be very effective in the context of COVID-19, wherein there were few cases relative to the number of travellers and many asymptomatic cases of the disease.

The only observational study included in the review investigated the effects of a comprehensive set of border control measures in Hong Kong alongside other NPIs that were all implemented between January and March 2020. The border control measures had been progressively adopted in the region, starting with the closure of border control points with mainland China in late January. This was followed by mandatory 14-day quarantine for inbound travellers, which first applied to just travellers from the mainland but eventually applied to all inbound travellers by mid-March. However, as all these measures were implemented around the same time, it was not possible to independently assess the effectiveness of border control measures in this context.

Given the consistency of the findings identified in this review, it seems likely that the border control measures imposed in the early phase of the epidemic (e.g. before March) had some impact on delaying the export of cases out of China and, thus, likely delayed the establishment of outbreaks outside of China, but clearly they were insufficient to stop the pandemic. All the identified studies found that the measures imposed on Wuhan travellers by Chinese officials were likely very effective in reducing exported cases and that the additional targeted measures against Chinese travellers by other countries played some role, but both types of measures became relatively ineffective within a few weeks as other places became the dominant source of imported cases.

Given the rapid review approach and the large number of modelling studies, this study opted to use a proprietary tool to assess the quality of the included studies. All but six of the studies included in this review suffered from a high or medium risk of bias because they did not adequately account for the impact of other measures in their evaluation of the effectiveness of border control measures.

### (b) Burns *et al.* [4]

This second rapid review also aimed to assess evidence related to travel-related controls implemented during the early phases of the COVID-19 pandemic by reviewing evidence from studies published or preprinted through 26 June 2020. It also included studies that investigated diseases other than COVID-19, but we did not focus on these findings in our synthesis. This review identified 17 modelling and eight observational COVID-19 studies. The review examined only international border control measures. It included studies that investigated full or partial border closures, entry and exit screening, and the quarantine of travellers.

The review identified 12 studies (11 modelling studies and one observational study) that examined the impact of international travel restrictions at the beginning of an outbreak, including studies from other international contexts such as the USA. It was found that these additional measures may have reduced the number of imported cases by 26–90% and delayed the time to an outbreak by up to 26 days. It should be noted that there is low confidence in the precision of these figures. Since many of these studies did not account for Wuhan measures, they likely overestimated the true effectiveness of the measures.

The review also identified 12 studies (six modelling studies and six observational studies) on screening with or without quarantine. Since this review comprised mainly studies from the early stage of the pandemic, all but two of the studies evaluated symptomatic screening strategies. The modelling studies generally assumed the effectiveness of the screening strategies in their evaluations based on assumptions on the proportion of cases that were symptomatic and could be identified by the screening methods. Nine of the studies investigated the number or proportion of infected travellers detected, while only three studies (all modelled) investigated whether or not the implementation of screening made a difference to domestic transmission. Based on these last three studies, the authors of the review concluded that screening might affect outbreak dynamics, but since this was based only on a small number of studies and the overall quality of evidence was low, they believed that the evidence on screening should be interpreted with caution. In contrast to the other measures evaluated, quarantine for periods of up to two weeks was found by one modelling study to result in large and measurable decreases in the probability that a case would enter the community and thus lead to onward transmission.

The review used the Quality Assessment of Diagnostic Accuracy Studies (QUADAS-2) tool to assess the risk of bias of observational studies evaluating the real-world performance of entry and exit screening (similar to diagnostic studies), the Risk Of Bias In Non-Randomized Studies of Interventions (ROBINS-I) tool for observational studies evaluating the impact of measures, and a bespoke tool for modelling studies. At the individual study level, the authors noted several sources of meaningful bias for the studies included in the review. The review also assessed the overall certainty of the evidence using the Grading of Recommendations Assessment, Development and Evaluation (GRADE) approach, and observed low to very low certainty across types of border control measures and outcomes.

In summary, similar to the previous review, this study found that travel restrictions were more effective than screening measures and that travel measures implemented in the early phase likely had some effect in limiting the initial spread of the virus outside China. Quarantine demonstrated promise; however, this was based on only a single identified study.

### (c) Bou-Karroum *et al*. [3]

Building on the growing corpus of effectiveness studies published by the end of 2020, this review adopted a search strategy like that of Burns *et al*. [4] but focused only on COVID-19 studies and included subnational studies. Therefore, it included most of the studies identified in the two previous reviews but identified more (*n* = 15) observational studies than those reviews. While this review also looked at non-epidemiological outcomes, we exclude them from our narrative synthesis as these outcomes are beyond the scope of this study. Unlike previous reviews, this review grouped all forms of travel restrictions with border closures and labelled them border closure policies, making its terminology inconsistent with that of the other reviews. We did not use this method to group the underlying studies identified in this review.

Despite the larger number of studies (32 of the studies included in this review had not been reviewed in either of the earlier reviews), the overall findings of this review were consistent with the previous reviews, concluding that some forms of border control measures contributed to reducing the number of cases, slowed the progression of outbreaks and reduced spread across jurisdictions. It also found that such measures are likely to be more effective when implemented early and when coupled with measures to reduce transmission in both the exporting and the importing regions.

Based on a larger number of studies that investigated the use of quarantine, it was found that the use of such measures can be highly effective in reducing transmission in countries implementing the measures. However, the studies generally used very long periods (i.e. up to 14 days), and thus the review concluded that such periods were required to achieve significant reductions in transmission. It also found that quarantines were even more effective when coupled with ‘pre-release’ testing, especially for shorter (i.e. seven-day) quarantine periods.

The review also investigated the use of symptomatic screening measures and, similar to the previously described reviews, concluded that the use of such measures is unlikely to be very effective in detecting a sufficiently large proportion of infected cases; however, the underlying studies suggest that symptomatic screening methods could be improved with more sensitive screening methods, awareness of travellers, asymptomatic testing, and exit screening in the country with the initial outbreak.

Using the GRADE tool for observational studies and the EVIDEM framework for modelling studies, the review found that there was a high risk of bias in about half of the observational studies due to the risk of confounding with other measures. The completeness of data also led to a high risk of bias in 73% of the observational studies. Most of the modelling studies lacked sensitivity analysis of their findings.

In summary, despite the larger number of studies, the findings of this study did not substantially differ from those of the two previous reviews, but the consistent findings seem to lend more credibility to the emerging consensus that travel restrictions in the early phase of the pandemic did have some effect and that quarantine, alone or in combination with diagnostic testing, appeared to be the most effective border control measure.

### (d) Burns *et al*. [5]

This updated review synthesized evidence from 62 studies (25 unique to this review) and was conducted by the same research group as in 2020. This time, they focused exclusively on evidence from COVID-19 but otherwise used the same methodology. Again, most studies were modelling studies (*n* = 48), but in this review many of the studies were contextualized to more diverse geographical contexts that were no longer aimed at the initial containment of the virus within China.

The main findings remained relatively consistent, although a larger number of studies allowed for a more nuanced understanding of effectiveness. Despite the large number of studies, all 31 studies that investigated the effectiveness of travel restrictions were modelling studies. Primarily, the review found that studies evaluating the effectiveness of travel restrictions showed that these measures had a positive effect in terms of the number of cases avoided, although a small number of studies found no or mixed effects. For example, 10 out of 13 studies that looked at whether travel restrictions reduced the number or proportion of cases in the community found that they did have a positive effect. However, even for this outcome there was wide variation in the estimated reduction in cases—from 1.8% up to 97.8%, depending on factors like the level of community transmission and the implementation of other NPIs.

Studies also investigated the use of screening, although a larger proportion of studies now focus on the use of diagnostic screening (i.e. testing of travellers via PCR), likely due to the increased use of such measures globally towards the end of 2020. Importantly, most studies also assumed that positive cases would be isolated from the public for a period (which differed across studies) to prevent onward community transmission. Symptomatic or exposure-based screening was again found to be less effective than diagnostic testing, although the studies in this review provided more support for the use of such measures than previous reviews. Diagnostic screening was again seen as a superior form of screening, and the five studies that investigated this intervention found that testing could detect 50–90% of cases, but this was very contextually specific.

Twelve new studies, all modelling, investigated the effectiveness of quarantine. All found some usefulness of quarantine but differed in terms of the number of cases avoided and/or detected. Notably, the level of compliance with the measure was seen as an important determinant of effectiveness, with mandatory facility-based forms of quarantine likely to be more effective than other quarantine modalities.

The combined use of testing and quarantine was evaluated separately. Notably, four observational studies found very high detection rates (68–92%) when these measures were combined, and the success varied according to the actual length of quarantine used and the frequency of testing employed.

As for [4], this review applied QUADAS-2 to assess the risk of bias of observational studies evaluating the real-world performance of entry and exit screening, ROBINS-I for observational studies evaluating the impact of measures, and a bespoke tool for modelling studies. Like the previous review, the authors identified and described several issues across all study designs that suggest a risk of bias of the included studies. Likewise, the certainty was rated either very low or low for all bodies of evidence except for one, which was rated moderate.

In summary, the review reinforced the view that some forms of travel restrictions could be effective, but that symptom-based screening was unlikely to be effective enough to warrant implementation. It also reinforced the view that quarantine of incoming travellers is likely to be among the most effective measures, especially when combined with diagnostic screening. The existence of observational data to support this conclusion is noteworthy. However, the number of studies was too small to draw firm conclusions regarding the optimal length of quarantine or testing modalities.

### (e) Hohlfeld *et al*. [7]

This review, which used the same search strategy as Burns *et al*. [5], describes itself as a companion review to the previous review and provides an update on the literature up to 6 June 2022. However, it focused on only a subset of border control measures, specifically quarantine, testing, and a combination of quarantine and testing. It identified 17 new studies, eight of which were observational.

The review found potentially high levels of effectiveness of quarantine, but the level of effectiveness varied according to the length of quarantine and levels of compliance. It was found that pre-departure microbiological (i.e. PCR-based) testing could reduce the risk of transmission during travel by 10–72%, depending on the length of time between the test and the date of travel. Finally, as in the previous reviews, the highest level of effectiveness was found when quarantine was combined with some form of diagnostic-based screening.

The overall risk of bias was deemed to be critical and moderate in eight studies, and the review generally found very low certainty of evidence across all the studies investigated. In short, this review did not substantially change the emerging consensus in the literature on the effectiveness of measures but provided more evidence to support this perspective.

### (f) Burns *et al*. [8]

Given that this review update is currently being finalized, the following are tentative findings and are subject to modification. This update largely followed the same methodology as the previous update [5]. A key difference is that modelling studies were considered for the data synthesis only in cases where they addressed what the authors labelled ‘priority policy questions'. These were questions relating to how variants of concern and/or population immunity due to COVID-19 vaccination coverage and/or previous infection influenced the effectiveness of border control measures, as well as how previous COVID-19 vaccination and/or infection was used as a screening measure.

This updated review identified and included 60 new studies since the previously published update, including 41 observational studies and 19 modelling studies addressing priority policy questions. Like the previous update, despite the growing evidence base, the overall findings around the effectiveness of travel measures remain largely unchanged, but this update does allow for a more nuanced exploration of some aspects of effectiveness.

For example, several new studies assessed the value of repeated testing of arriving travellers at various intervals during quarantine, providing more support for the view that such strategies can shorten the required quarantine time without leading to a meaningful increase in imported infections. Specifically, four observational studies of real-world national border control measures found that countries which implemented a 14-day quarantine with multiple testing [10,11] and countries that implemented a 7- or 8-day quarantine with multiple testing [11–13] were both able to detect around 80–95% of cases. We interpret this to mean that when implemented with multiple testing, an additional week of quarantine did not lead to a much larger proportion of cases detected. This would be consistent with studies of the mean generation time of the SARS-CoV-2 virus, which has been estimated at various times during the pandemic to range from 2.3 to 7.5 days and to become shorter over time with the emergence of new variants [14,15].

Other notable findings include that history of COVID-19 vaccination and/or infection, when used as a screening measure in four studies, generally performs better than other screening measures in the short term for preventing infected individuals from crossing borders. The review also identified some modelling studies assessing how variants of concern or population immunity due to COVID-19 vaccination coverage and/or previous infection influenced the effectiveness of border control measures. These studies appear to suggest, as expected, that border control measures will be less effective for more transmissible variants of concern. Some modelling studies also suggest that increasing population immunity in both the travelling and the local populations decreases the number of potentially infectious individuals, thus reducing the value of border control measures over time.

### (g) A forward citation search based on Burns *et al*. [5]

As of 28 February 2023, this rapid review was cited 75 times. Of the studies that cited this review, only three fit the original eligibility criteria and had been published since the most recent searches by the author team. Two of these studies represent novel additions to the evidence base, as they evaluate the effectiveness of screening measures for avoiding the importation of cases of the Omicron variant. However, from these two studies it does not appear to be possible to concretely assess whether these measures were more effective for the Omicron variant than for previous variants.

## 4. Discussion

This narrative synthesis of the existing systematic review evidence was motivated by the desire to better understand what has been learned about the effectiveness of the various types of border control measures adopted during the COVID-19 pandemic, specifically the types of border control measures, geographical contexts and times at which these measures were most effective. Overall, the literature is consistent with several findings regarding the effectiveness of specific types of measures.

First, symptomatic or exposure-based screening measures, which were among the first measures widely adopted by many countries in early 2020, were not effective enough to have had a meaningful effect on reducing importations and transmission. However, the evidence also suggests that diagnostic-based screening measures, usually in the form of pre-departure or upon-arrival PCR-based testing, increased the predictive power of screening. But at best such testing alone is unlikely to detect more than about half of potentially infected travellers. More recent studies suggest that screening based on vaccination or recent infection status was potentially even more effective than diagnostic testing in preventing importation and onward transmission.

Second, the types of targeted travel restrictions levied against Chinese travellers in early 2020 likely had an immediate effect on reducing transmission, but they quickly became less effective as other jurisdictions became the major source of cases. A similar narrative emerged around targeted travel restrictions levied on travellers from Iran, South Korea and Italy, although there was less evidence based on the effectiveness of these measures. Most studies also concluded that earlier implementation generally led to higher levels of effectiveness.

Third, the evidence on quarantine consistently demonstrated that it had the highest levels of effectiveness of the single interventions evaluated; however, most evidence comes from long quarantine periods (e.g. 14 days). Studies have also consistently concluded that compliance with quarantines, which tended to be lower when quarantines were self-monitored, was an important determinant of their effectiveness. Also, the literature consistently found that when coupled with diagnostic testing, quarantines were more effective and could be shortened without a substantially increased risk of transmission. A study from Korea (not included in this review) suggested that up to 95% of symptomatic cases infected with the Omicron strain of the virus would go on to develop symptoms within six days [16], suggesting that shorter quarantine periods with appropriate testing could achieve the same level of risk reduction as lengthier quarantines.

Fourth, with regard to travel restrictions (whether full or partial border closures), much of the evidence of their effectiveness was sourced from studies of targeted measures aimed at travellers from initial hotspots (e.g. Wuhan, Iran, South Korea and Italy) during the initial containment stage. However, countries continued to implement these measures well after widespread community transmission was established in most international contexts, and evidence of the effectiveness of these measures at those time periods is very limited. Relatedly, there is limited evidence from the included systematic review studies on the effectiveness of later rounds of border control measures aimed at stopping or slowing the importation of new variants of concern, despite the widespread use of border control measures to try to slow the introduction of new variants. In addition, the widespread adoption of international border control measures as well as domestic mobility controls globally led to an overall reduction of travel, which itself likely led to a reduction in imported cases, even in locations without border control measures in place. Similarly, border control measures adopted in one country may have effects in other countries, as travellers who would have otherwise travelled to the adopting country went elsewhere. Few studies adequately accounted for the broader effects of the pandemic and pandemic response measures in deterring or displacing international travel.

Fifth, the evaluation of comprehensive border control regimes, such as those that enabled some places to maintain zero-COVID status into 2021 (e.g. Singapore and New Zealand) or 2022 (e.g. Hong Kong and mainland China), was largely excluded from existing systematic reviews because of the inclusion criteria based on the evaluation of specific border control measures. For example, in Hong Kong border control measures first began as targeted restrictions but eventually escalated into a border control regime that included mandatory quarantine for all inbound travellers, extensive pre-, upon- and post-arrival testing, and strong domestic policies to provide surveillance to detect any breakthrough cases [17]. It is very difficult to independently evaluate the contribution of each of these measures given that many were implemented around the same time or at times in which there was limited domestic transmission owing to the use of previous NPIs. Moreover, strong domestic NPIs were also necessary to maintain such elimination status; not all the ‘success’ of these regions can be attributed to international border control measures. Nonetheless, overlooking these cases may potentially miss important lessons about the effectiveness of border control measures, and border controls, as part of a broader package of measures, were used in places which successfully maintained zero-COVID status for many months after the start of the pandemic.

Notably, the types of measures implemented, and thus evaluated, changed over the duration of the pandemic. This might have resulted from policymakers striving to achieve different outcomes with their border control measures over the duration of the pandemic (including outcomes unrelated to transmission, though these are beyond the scope of this study). For example, initially the goal may have been to prevent, or at least delay, the importation of any cases, which could have prevented the establishment of COVID-19 in their jurisdictions. Once community transmission had been established, however, it might be that the goal became to reduce the number of imported cases so that they did not further contribute to domestic epidemic dynamics. Similarly, once a high level of population immunity was achieved through infection or vaccination, the value of border control measures likely decreased. Later, rounds of measures were adopted in response to the rise in the Delta and Omicron variants, as well as the re-opening of mainland China, in hopes of preventing the importation of new variants of COVID-19. In addition, some countries aimed to achieve complete local elimination (i.e. ‘zero-COVID’) whereas others clearly did not. These varied goals mean that applying a single definition of effectiveness can be problematic. While some modelling studies explored these aspects of effectiveness, the literature, and thus the associated reviews, does not provide great insights into some of these important contextual questions.

While the studies included in these reviews were conducted in a diverse set of countries, there is still very limited evidence from many parts of the world. We note that there were many studies from places such as Australia, Hong Kong, Singapore and Japan, where more robust measures had been put into place and where better data to evaluate these measures existed; however, there were fewer studies from other areas (e.g. Central/South American and African countries). The inclusion of more or higher-quality studies from countries with more robust measures might bias the overall findings towards higher levels of effectiveness.

The examined systematic reviews point to several inherent challenges in the evaluation of border control measures. Border control measures were rarely the only control measures adopted by countries during the pandemic, and few studies adequately controlled for these other interventions, likely leading to bias in their estimates of effectiveness. Only a few studies leveraged quasi-experimental study designs, which, in principle, should better control for both observed and unobserved confounders. Specific designs included, for example, the controlled interrupted time-series design [18], the synthetic control design [19] and the event study design [20]. Moreover, most of the border control measures implemented were temporary measures and oftentimes countries continuously changed the specific regulations in place, which further complicated their evaluation.

In addition, the large number of modelling, versus observational, studies is another important limitation of the existing literature. Modelling studies represent abstractions of the real world, and the extent to which they reflect reality is unclear. The quality of modelling studies depends on the underlying models and assumptions used, and it is difficult to evaluate the quality of such studies in practice. On the other hand, observational studies draw on real-world data but often use study design and statistical methods that make interpretation and use of their findings challenging. Many observational studies, for example, employed ecological methods to conduct comparisons across several countries, many of which likely have very different underlying systems for tracking COVID-19 and/or very different systems for designing, implementing and enforcing border control measures. Additionally, these and other studies often involved the use of composite measures for border controls, such as the Oxford COVID-19 Government Response Tracker, rather than specific real-world border control measures; such composite measures run the risk of lumping together and comparing different types of border control measures. Many observational studies have evaluated screening and quarantine measures in a way that did not reflect large national-level policies. Only a few studies evaluated real-world border control programmes involving hundreds of thousands of travellers over extended periods of time, such as the notable studies conducted in Qatar [21] and the UK [22].

The use of the systematic review methodology also meant that many studies did not meet the eligibility criteria of the examined reviews but nevertheless can provide important insights into the effectiveness of border control measures. For example, studies of quarantine failure, which is not an intervention per se, in Australia, New Zealand and Hong Kong provide interesting insights into the operational challenges of maintaining these strict border control regimes for international travellers [23,24].

Also, while all observational primary studies included in the reviews drew from data on observed COVID-19 cases, phylogenetic data on the introduction of specific viral lineages in relation to the timing of border control measures can also be insightful. For example, one study found that more genetically distinct lineages in the UK had been imported from countries without a mandatory 14-day quarantine period than from countries not subject to these measures after they were introduced in the summer of 2020 [25]. Another study found that the strictest forms of border control measures used in Switzerland led to an 86–98% reduction in lineages from abroad [26]. Travel restrictions introduced in March 2020 may have limited the number of lineages of the virus in Ukraine [27]. Another study found that most of the lineages that later dominated the Delta variant wave had been introduced into the UK prior to the implementation of hotel quarantine for incoming travellers [28]. Targeted travel restrictions against travellers from southern African countries were largely ineffective at reducing the number of lineages of the Omicron variant in the UK, owing to the fact that many lineages had already been imported by the time the measures were put into place and because the USA quickly became an important source of introductions and travellers from there were not subject to any form of control measure at the time [29]. There was also limited evaluation of the impact of border control measures on equity or other considerations, which is another important limitation of the existing literature.

Border control measures can be among the most economically costly of the COVID-19 control measures adopted during the pandemic. For example, evidence suggests that cross-border and informal trade account for 40% of the gross domestic product and 55.7% of employment in sub-Saharan Africa [18]. Given that nearly every country implemented some form of international border control measure during the first few months of the pandemic and there were very large reductions in international travel, it is important that we have a clear understanding of the extent to which these measures contributed to a reduction in global transmission. This synthesis suggests that, on balance, some border control measures were more effective than others, in particular quarantine, but a great deal about the context and implementation of these measures remains poorly understood. Additionally, given the high costs of these measures, simply showing that they work may not be sufficient to justify their use, since other, less economically costly NPIs—such as social distancing or mask wearing—may be at least as effective as measures imposed at the border. Given their widespread adoption and sustained use over the pandemic, it seems that many policymakers believe that the benefits of these measures outweigh their costs.

When the WHO declared the COVID-19 outbreak a PHEIC (public health emergency of international concern), which it did with the authority granted to it under the International Health Regulations [30], it did not recommend the use of any international border control measures, citing limited evidence of their effectiveness. This synthesis, as well as the underlying systematic reviews, does question this initial assumption and provides evidence that in some circumstances, some forms of border control measures were useful in affecting transmission; however, much remains uncertain. Thus, continuing to learn about what types of studies have been more useful than others during the COVID-19 pandemic, and may offer useful insights for future infectious disease outbreaks, should continue to be a priority for public health researchers.

## Data Availability

A form of the data will be included in the published article.
